# Non-Rigid Cycle Consistent Bidirectional Network with Transformer for Unsupervised Deformable Functional Magnetic Resonance Imaging Registration

**DOI:** 10.3390/brainsci15010046

**Published:** 2025-01-05

**Authors:** Yingying Wang, Yu Feng, Weiming Zeng

**Affiliations:** Lab of Digital Image and Intelligent Computation, College of Information Engineering, Shanghai Maritime University, Shanghai 201306, China; wangyingying0057@stu.shmtu.edu.cn (Y.W.); fyuchn@163.com (Y.F.)

**Keywords:** fMRI, image registration, deep learning, unsupervised, Transformer

## Abstract

Background: In neuroscience research about functional magnetic resonance imaging (fMRI), accurate inter-subject image registration is the basis for effective statistical analysis. Traditional fMRI registration methods are usually based on high-resolution structural MRI with clear anatomical structure features. However, this registration method based on structural information cannot achieve accurate functional consistency between subjects since the functional regions do not necessarily correspond to anatomical structures. In recent years, fMRI registration methods based on functional information have emerged, which usually ignore the importance of structural MRI information. Methods: In this study, we proposed a non-rigid cycle consistent bidirectional network with Transformer for unsupervised deformable functional MRI registration. The work achieves fMRI registration through structural MRI registration, and functional information is introduced to improve registration performance. Specifically, we employ a bidirectional registration network that implements forward and reverse registration between image pairs and apply Transformer in the registration network to establish remote spatial mapping between image voxels. Functional and structural information are integrated by introducing the local functional connectivity pattern, the local functional connectivity features of the whole brain are extracted as functional information. The proposed registration method was experimented on real fMRI datasets, and qualitative and quantitative evaluations of the quality of the registration method were implemented on the test dataset using relevant evaluation metrics. We implemented group ICA analysis in brain functional networks after registration. Functional consistency was evaluated on the resulting t-maps. Results: Compared with non-learning-based methods (Affine, Syn) and learning-based methods (Transmorph-tiny, Cyclemorph, VoxelMorph x2), our method improves the peak t-value of t-maps on DMN, VN, CEN, and SMN to 18.7, 16.5, 16.6, and 17.3 and the mean number of suprathreshold voxels (*p* < 0.05, t > 5.01) on the four networks to 2596.25, and there is an average improvement in peak t-value of 23.79%, 12.74%, 12.27%, 7.32%, and 5.43%. Conclusions: The experimental results show that the registration method of this study improves the structural and functional consistency between fMRI with superior registration performance.

## 1. Introduction

Functional magnetic resonance imaging (fMRI) is extensively utilized in medical imaging research. Neuronal activity in the human brain will cause changes in blood oxygen concentration, and fMRI can noninvasively measure changes in blood oxygen levels over time, which can help to study the function and mechanism of the human brain. A series of preprocessing steps are required before statistical analysis of fMRI from a group of subjects, including a key step of spatial normalization. Due to the differences in age, gender, and health condition, subjects have different spatial locations, sizes, and shapes. Therefore, it is necessary to conduct spatial standardization of fMRI from different individual spaces. Accurate and effective registration helps to improve the statistical capacity of group analysis among subjects.

fMRI registration is usually divided into one-step and two-step registration. The one-step registration refers to directly registering individual functional images to a standard functional image template [1,2]. The two-step registration first performs an affine transformation between fMRI and structural MRI, and then non-linearly distorted structural MRI to a common standard spatial structural template [1], and then applies the warp parameters to fMRI. There are two standard spatial templates commonly used: the MNI template and the Talairach template [3]. Registration methods for structural images include linear and nonlinear transformation methods [4]. Linear transformation methods mainly use translation, rotation, and scaling. Nonlinear transformation, also called elastic and deformable transformation, implements transformations for each voxel. Classical nonlinear transformation algorithms include Demons [5], LDDMM [6], and DARTEL [7]. For each subject, structural MRI has high spatial resolution and clear anatomical structures. Therefore, traditional fMRI spatial normalization used to be achieved through structural MRI registration. However, it has been shown that this method cannot achieve accurate functional consistency [8,9].

Some researchers proposed Bayesian functional registration for fMRI [10,11], combined with the Bayesian framework, to evaluate the brain functional and activation differences and achieve functional feature alignment. Pu et al. maximize the mutual information between images and perform rigid registration of fMRI by iterative optimization [12,13]. More accurate non-rigid registration is needed to achieve better registration performance. Wang et al. proposed customized fMRI registration templates, which were constructed using time series fusion [14]. Chen et al. utilized the functional network of the brain to establish the correspondence between images and register fMRI to the common space [15]. Khullar et al. proposed a method for spatial normalization of fMRI data using group ICA network to achieve function normalization [16]. Some researchers proposed fMRI registration methods based on global functional connectivity patterns [9,17,18]. Jiang et al., respectively, proposed fMRI alignment based on local functional connectivity patterns [19,20,21]. Most of these methods utilize functional connectivity features on the highly curved gray matter cortex for registration, ignoring the importance of the white. Zhou et al. proposed an fMRI registration method with tissue-specific patch-based functional correlation tensors [22]. The deformation field between images is estimated by extracting the functional correlation tensor features from gray matter and white matter, and then the mLDDMM is applied to achieve the registration. These traditional registration methods based on iterative optimization are time-consuming and inefficient. Freire et al. proposed a fMRI 3D registration method using neural networks [23]. The method extracts subsets from Fourier space and learns six registration parameters for 3D rigid transformation through six neural networks, including three translation parameters and three rotation parameters. The rigid registration method does not achieve accurate nonlinear registration.

In recent years, deep learning methods have been gradually applied to fMRI registration, which can automatically learn the features of the images and greatly improve registration efficiency. Deep learning registration can be divided into semi-supervised/supervised registration and unsupervised registration. Supervised deep learning registration often needs to provide ground truth deformation fields as supervisory information to assist registration [24,25,26,27]. Its performance is often limited due to the difficulty of obtaining realistic deformation fields because it relies on ground truth voxel mappings, which are difficult to obtain since there are few individuals with expert knowledge to perform fMRI registrations [28]. Zhu et al. proposed a semi-supervised deep learning model for fMRI registration by adding supervised information [28,29]. Unsupervised deep learning registration methods overcome the lack of real deformation field datasets, trained by minimizing the similarity loss between the registered and fixed image [30,31,32,33]. Lin et al. proposed an unsupervised cascaded network for fMRI registration [34,35]. It has been proven that unsupervised deep learning methods can achieve excellent fMRI registration performance, which is promising.

In this paper, we proposed a non-rigid cycle consistent bidirectional registration network with Transformer for unsupervised deformable fMRI registration. The main research and contributions of this work are as follows:

Usually, deep learning-based fMRI registration methods use convolutional neural networks, and the effective receptive field of the network is limited. To address the issues, we apply the Transformer in the registration network to establish remote spatial mapping between image voxels to improve the ability to extract image features. In the method, fMRI registration is performed based on the structural MRI, the deformation field of the structural MRI is downsampled to obtain the deformation field of fMRI, and the local functional connectivity information of fMRI is added during training to improve the functional consistency of fMRI images. To reduce the spatial folding of the deformation field and maintain the topology during deformation, this study considers bidirectional image registration and implements forward and reverse registration for image pairs. The final registration performance can achieve accurate structural consistency and functional consistency between fMRI. Furthermore, it greatly improves the computational efficiency of fMRI registration.

## 2. Materials and Methods

### 2.1. Local Functional Connectivity Pattern

Functional connectivity (FC) represents the functional connections that exist between different brain locations and is usually measured by the correlation of time series between (regions of interest) ROIs. FC is an important functional feature of fMRI, which can be used as functional information for fMRI registration to improve functional correspondence [17,18]. According to the size of the spatial neighborhood, FC can be divided into global functional connectivity (GFC) and local functional connectivity (LFC). GFC focuses on the functional connectivity between a certain ROI and all other ROIs in the brain. LFC focuses on the functional connectivity of a certain ROI with ROIs within its local spatial neighborhood. It has been shown that GFC is sensitive to the spatial location of ROIs in registration and does not have good robustness, and smaller spatial neighborhoods have better robustness for fMRI registration [20]. In this paper, the LFC pattern is utilized as the functional information of fMRI.

The time series at each voxel *p*, including all time points, can be regarded as a vector value *I(p)*. The FC between two voxels is generally computed by the Pearson correlation coefficient, calculated as

(1) $$C\left(p,q\right)=\frac{<I\left(p\right)-\bar{I\left(p\right)},\;I\left(q\right)-\bar{I\left(q\right)}>}{\left||I\left(p\right)-\bar{I\left(p\right)}>||\cdot ||I\left(q\right)-\bar{I\left(q\right)}>|\right|},$$

where $\bar{I\left(p\right)}$ *(*$\bar{I\left(q\right)}$) denotes the vector mean of the time series at voxel *p (q)*. The LFC designed in this study is the functional connectivity within the local spatial neighborhood at each voxel.

For voxel *p*, set its local spatial neighborhood as a square spatial neighborhood with the size of *n* voxels. The LFC pattern is calculated as the set of FC values between the voxel and all voxels in its local spatial neighborhood. The local functional connectivity information at this voxel can be represented by its local functional connectivity pattern. For the local spatial neighborhood, the size and shape of the local spatial neighborhood at each voxel may be fixed or varied. In this paper, we mainly use the local functional connectivity pattern with fixed neighborhood size. Regarding the neighborhood size, this study refers to the hyperparametric experiment conducted by Li et al., which concluded that the best registration is achieved when the square neighborhood size is w = 21 [29]. In this study, a square neighborhood of 3 voxels is used when extracting the functional information.

In the process of extracting the local functional connectivity patterns at voxel, due to the selection of calculation order and the influence of spatial relative position when calculating the functional connectivity between each pair of voxels, the local functional connectivity patterns calculated each time are random and unstable. To improve the stability of the extracted local functional connectivity patterns, we express the extracted local functional connectivity patterns at each voxel in the form of the probability distribution, eliminating the unstable effects caused by the calculation order and spatial relative position. We chose the kernel probability density estimation to estimate the probability distribution of localized FC patterns. We obtain a series of sampling points in the interval [−1, 1] by uniform sampling of FC values. For the set of LFC values at voxel p, we use kernel density estimation (KDE) to estimate the probability density at the uniform sampling points. The set of probability densities at all sampling points is represented as a feature vector $f\left(I\left(p\right)\right)$. The kernel density estimation formula is as follows:

(2) $${\hat{f}}_{h\left(x\right)}=\frac{1}{n}\sum_{i=1}^{n}K_{h}\left(x-x_{i}\right)=\frac{1}{nh}\sum_{i=1}^{n}K\left(\frac{x-x_{i}}{h}\right),$$

Specifically, using the Gaussian kernel as the kernel function for kernel probability density estimation,

(3) $$K\left(x\right)=\frac{1}{\sqrt{2\pi }}exp\left(-\frac{1}{2}x^{2}\right),$$

The probability density of the connection value at the nth sampling point is calculated as

(4) $$\begin{array}{c} f^{n}\left(I\left(p\right)\right)=\frac{1}{N}\sum_{q\in N\left(p\right)}K_{h}\left(n-C\left(p,q\right)\right),\\ =\frac{1}{N}\sum_{q\in N\left(p\right)}\frac{1}{(2\pi h^{2}{)}^{\frac{1}{2}}}exp\{-\frac{{\left||n-C(p,q)|\right|}^{2}}{2h^{2}}\} \end{array}$$

where $K_{h}$ is a Gaussian kernel with standard deviation *h*, and $N\left(p\right)$ is the number of voxel points in the local spatial neighborhood at voxel p. The LFC pattern at voxel point *p* is finally expressed as a feature vector $f\left(I\left(p\right)\right)$,

(5) $$f\left(I\left(p\right)\right)=\left[f^{1}\left(I\left(p\right)\right),f^{2}\left(I\left(p\right)\right),\ldots ,f^{n}\left(I\left(p\right)\right)\right],$$

### 2.2. Cycle Consistent Bidirectional Network

This study considers bidirectional image registration and implements forward and reverse registration for image pairs. The deformation field generated by the general unsupervised learning image registration method is directional; for example, from the moving image to the fixed image, only a single direction is registered. This unidirectional deformation field is not reversible, and the continuous deformation image obtained by using the inverse deformation field to deform the registered image cannot be easily restored to the original image. Therefore, the deformation field generated by the general one-way registration method tends to produce a large spatial folding, which makes the deformation field not smooth and fails to maintain topology well. Most of the image registration methods do not consider the deformation field reversibility, and the quality of the deformation field is not high. To reduce the spatial folding of the deformation field and maintain the topology during deformation, we designed the unsupervised deformable fMRI registration network with non-rigid cyclic consistent bidirectional registration.

**Figure 1:**
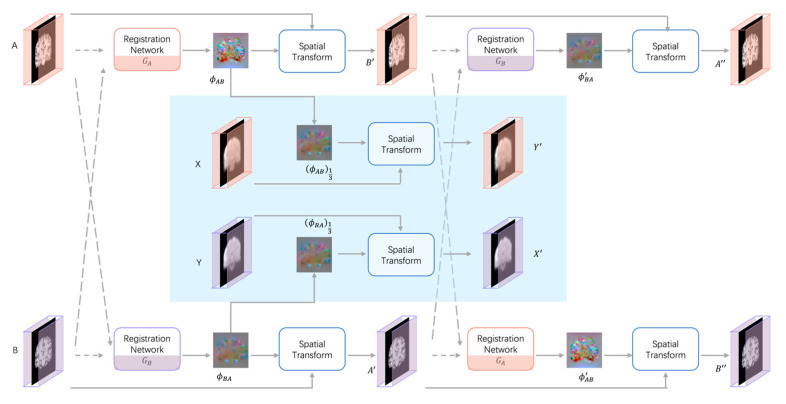
The overall framework of the proposed non-rigid cycle consistent bidirectional network for unsupervised deformable fMRI registration. Short and long dashed lines represent the moving and fixed images, respectively.

The overview of the proposed unsupervised deep learning network for fMRI registration is shown in Figure 1, it is a cyclic consistent bidirectional registration network on the whole. The network takes four inputs: MRI images (A, B); fMRI images (X, Y), A is the structural MRI corresponding to X, and B is the structural MRI corresponding to Y. A and B and X and Y have been registered by affine alignment. The two registration networks ($G_{A}$, $G_{B}$) denote forward and reverse registration, respectively. $G_{A}$: (A → B) → $\phi_{AB}$ represents the registration network from A to B, which generates the deformation field $\phi_{AB}$ from A to B; $G_{B}$: (B → A) → $\phi_{BA}$ represents the registration network from B to A, which generates the deformation field $\phi_{BA}$ from B to A. The MRI images A and B are taken as moving and fixed image, respectively, and input into the forward registration network $G_{A}$ to generate the deformation field $\phi_{AB}$. The MRI images B and A are taken as moving and fixed images, respectively, and input into the inverse registration network $G_{B}$ to generate the deformation field $\phi_{BA}$. The deformed images $B^{’}$, $A^{’}$ are generated by the spatial transformer networks (STN)[36] according to the corresponding deformation field. The deformed images $B^{\prime }$, $A^{\prime }$ are used as the input of the network again to obtain the continuous deformed image by ensuring the continuous deformed image is restored to the original image, making the network cyclically consistent. Meanwhile, the deformation fields $\phi_{AB}$ and $\phi_{BA}$ obtained from MRI registration are downsampled and obtain the deformation fields ${\left(\phi_{AB}\right)}_{\frac{1}{3}}$ and ${\left(\phi_{BA}\right)}_{\frac{1}{3}}$. Then, fMRI images X and Y are taken as moving and fixed image, respectively, and the moving images X are deformed by the deformation field ${\left(\phi_{AB}\right)}_{\frac{1}{3}}$ through the spatial transformer networks to obtain the registered fMRI image $Y^{’}$. In the same way, the fMRI image Y and X are taken as the moving and the fixed image, respectively, and the moving image Y is deformed by the deformation field ${\left(\phi_{BA}\right)}_{\frac{1}{3}}$ through the STN to obtain the fMRI image $X^{’}$. The registration network is trained by minimizing the loss during the registration process. The continuous deformation image obtained after the original moving image is deformed by two reverse registration networks should be able to return to the original image under the condition with good reversibility of the deformation field. Imposing the cycle consistency constraint between the original image and the successively deformed image allows the networks to offer homeomorphic mappings capable of preserving topology [37].

### 2.3. Registration Network

Deep- learning-based image registration generally uses U-Net [38] as the registration network. By introducing the up-and-downsampling operation, the U-Net network expands the effective receptive field of the convolutional neural network and improves the modeling ability of the convolutional neural network for remote spatial information. With the continuous improvement of the U-Net network, some advanced U-Net network structures have been proposed and well applied in other medical image processing fields. However, limited work has been carried out in medical image registration using advanced U-Net network architecture.

Transformer has a favorable advantage in image registration; compared to the narrow and limited receptive field of convolutional neural networks, it has a larger receptive field and a self-attentive mechanism to better capture the spatial positional correspondences between images, focusing on the different spatial parts. This is in line with the purpose of image registration, which is to establish spatial correspondences between image pairs. Therefore, we consider adding Transformer to the U-Net network structure to improve the feature extraction capability in image registration.

**Figure 2:**
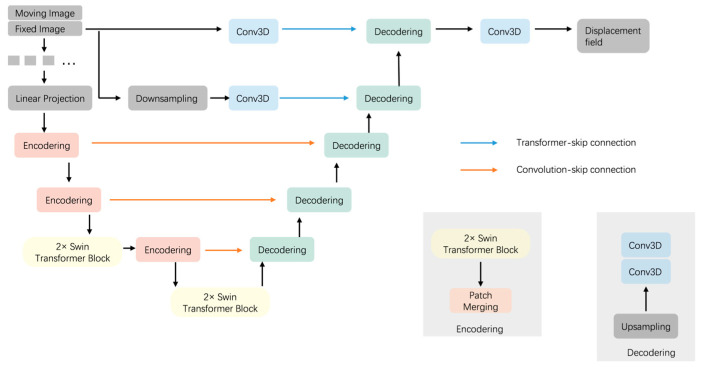
Structure of the proposed registration network.

As shown in Figure 2, we see the structure of the registration network in our proposed model. The registration network we use is improved on the network structure of U-Net by incorporating a local window attention mechanism (Swin Transformer) [39] in the encoder part and using a convolutional neural network in the decoder part. Specifically, our registration network is a U-Net-like network structure that mainly consists of a 6-layer encoder–decoder structure using skip connections between the encoder and decoder at each layer. A 2-channel 3D image stitched from the moving image $I_{moving}$ and the fixed image $I_{fixed}$ is first taken as the input, and then the stitched 2-channel image is segmented into non-overlapping blocks and sent to the encoder part. The spatial correspondence between images is better captured by segmenting the image into small patch blocks and using Swin Transformer in the encoding stage to compute the attention within a local window. The image feature mappings extracted after the coding layer are transferred to the corresponding decoding layer through skip connections to connect with the corresponding feature mappings of the decoding layer to reduce the loss of image information in the downsampling process. Finally, we output a dense nonlinear deformation field between the input image pairs.

Swin Transformer is a novel visual Transformer that can be used as a general backbone for computer vision tasks at the pixel level of high-resolution images such as object detection and image segmentation [39]. To adapt Transformers from natural language processing to computer vision, Swin Transformer constructs hierarchical feature maps based on the difference in scale between language processing and visual elements. As the network deepens, the feature maps are progressively downsampled in deeper layers through patch merging and reduce the resolution, resulting in hierarchical feature maps. In particular, Swin Transformer splits the image into non-overlapping patches of equal size depending on the window size and then calculates self-attention in non-overlapping windows locally.

The structure of two consecutive Swin Transformer blocks is shown in Figure 2. The first Swin Transformer block uses a window-based multi-head self-attention module (W-MSA). The second Swin Transformer block uses the shifted-window-based multi-head self-attention module (SW-MSA). These two Swin Transformer block structures are used in pairs in sequential order in practice.

In the 3D image, the window-based multi-head self-attention module (W-MSA) divides the feature maps of different resolutions into multiple windows according to a certain size ($M_{x},\;M_{y},\;M_{z}$) and then limits the self-attention to the local window range, which reduces the computational complexity and computational quantity compared with the multi-head self-attention module (MSA). In the process of calculating the self-attention, W-MSA adds relative position encoding. The calculation of self-attention within the local window is

(6) $$Attention\left(Q,K,V\right)=SoftMax\left(\frac{QK^{T}}{\sqrt{d}}+B\right)V,$$

where *Q*, *K*, and *V* are query, key, and value matrices belonging to $R^{M_{xyz}\times d}$, and d is the dimension of query and key. $M_{xyz}$ = $M_{x}\times M_{y}\times M_{z}$ is the number of patches in the 3D local window. Because the relative position values on each dimension (x,y,z) range from [-$M_{x,y,z}+1,M_{x,y,z}-1$], the bias matrix B representing the relative position information of the patches in each window can be set to a smaller size, such as $R^{\left(2M_{x}-1\right)\times \left(2M_{y}-1\right)\times (2M_{z}-1)}$.

Since W-MSA computes self-attention within non-overlapping local windows, neighboring windows lack connections. SW-MSA solves this problem through the shift window mechanism, which enables information exchange between different windows. In the case of 3D images, SW-MSA shifts the windows by ($\frac{M_{x}}{2},\frac{M_{y}}{2},\frac{M_{z}}{2}$) voxels based on the windows divided by W-MSA. For example, the input feature map voxel resolution is 4 × 8 × 8 and the window size is 2 × 4 × 4, the W-MSA module of the first Swin Transformer block divides the image into 2 × 2 × 2 = 8 windows. After the SW-MSA module of the second Swin Transformer block, the window is shifted by $\frac{2}{2}\;$× $\frac{4}{2}\;$× $\frac{4}{2}$ = 1 × 2 × 2 = 4, and the number of windows after shifting is 3 × 3 × 3 = 27 windows. The transfer of information between different local windows is easily realized by shifting windows.

The computation of two consecutive Swin Transformer blocks can be expressed as follows:

(7) $${\hat{z}}^{l}=W-MSA(LN{(z}^{l-1}))+z^{l-1},\;z^{l}=MLP\left(LN\left({\hat{z}}^{l}\right)\right)+{\hat{z}}^{l},\;{\hat{z}}^{l+1}=SW-MSA(LN(z^{l}))+z^{l},\;z^{l+1}=MLP\left(LN\left({\hat{z}}^{l+1}\right)\right)+{\hat{z}}^{l+1},$$

where ${\hat{z}}^{l}$ denotes the output features of the W-MSA module for block l; ${\hat{z}}^{l+1}$ denotes the output features of the SW-MSA module for the block l + 1; $z^{l}$ denotes the output features of the MLP module for the block l; and $z^{l+1}$ denotes the output $z^{l+1}$ of the MLP module for the block l + 1.

### 2.4. Spatial Transformer Networks

After the deformation field *ϕ* is predicted, the spatial transformation network STN [36], which can spatially deform $I_{moving}$ into $I_{fixed}$ according to *ϕ*, obtains the deformed image $I_{warped}$ ($I_{moving}\;$ ◦ *ϕ*). The grid generator module of STN generates a sampling grid according to the parameters of the deformation field, and then the sampler module samples the input image according to the generated grid. STN uses the trilinear interpolation method when sampling the voxels of the 3D image, taking the 8 voxels in the cube neighborhood at the voxel for interpolation. The calculation method for voxel *p* is as follows:

(8) $$I_{moving}◦\phi \left(p\right)=\sum_{q\epsilon N\left(p^{’}\right)}I_{moving}\left(q\right)\Pi_{d\epsilon \left\{x,y,z\right\}}\left(1-\left|p_{d}^{\prime }-q_{d}\right|\right),$$

where $p^{\prime }$ = *p* + *u(p)*. $N\left(p^{\prime }\right)$ denotes the 8 neighboring voxels around $p^{\prime }$; d denotes the three spatial dimensions. The above STN is calculated differentiably. During the training of the registration, we can calculate the gradient and make backpropagation errors, as well as learn the optimal parameters by minimizing the similarity difference between $I_{moving}\;$◦ *ϕ* and $I_{fixed}$.

### 2.5. Loss Functions

According to the energy function for iterative optimization in traditional image registration, the loss function for unsupervised image registration generally consists of two parts, the similarity measure loss between the registered and the fixed image and the smoothness loss to the deformation field, which can be expressed as

(9) $$L\left(I_{fixed},I_{moving},\phi \right)=L_{similarity}\left(I_{fixed},I_{moving}◦\phi \right)+\lambda L_{smooth}\left(\phi \right),$$

where λ is the regularization parameter. The overall loss function of our proposed unsupervised deep learning fMRI image registration network consists of four parts, which are structural MRI registration loss, functional MRI registration loss, cycle loss, and identity loss.

The structural MRI registration loss includes two parts: structural image similarity loss and deformation field smoothness loss. For the similarity loss between the image after registration and the fixed image, this study uses the often-used MSE function,

(10) $$L_{similarity}\left(I_{fixed},I_{moving}◦\phi \right)=MSE\left(I_{fixed},I_{moving}◦\phi \right)\;=\frac{1}{\left|\Omega \right|}\sum_{p\in \Omega }[I_{fixed}\left(p\right)-[I_{moving}◦\phi ](p){]}^{2},$$

where p denotes the voxel location in the image, and Ω denotes the image spatial domain.

In the image registration process, the image after registration becomes similar to the fixed image progressively, which will lead to spatial folding of the deformation field and unrealistic physical structure. To preserve the topology and smoothness of the deformation field, the spatial gradient of the displacement field is generally computed using L2 regularization to penalize the folding of the deformation field.

(11) $$L_{smooth}\left(\phi \right)=\sum_{p\in \Omega }{\left|\left|\nabla u\left(p\right)\right|\right|}^{2},$$

where $\nabla u\left(p\right)$ = ($\frac{\partial \phi \left(p\right)}{\partial x},\frac{\partial \phi \left(p\right)}{\partial y},\frac{\partial \phi \left(p\right)}{\partial z})$ denotes the spatial gradient of the displacement field u at the voxel *p*.

In the registration network of this study, the total loss of structural MRI registration can be expressed as

(12) $$L_{register\_mri}\left(A,B,G_{A,},G_{B}\right)=L_{register}\left(A,B,G_{A}\right)+L_{register}\left(B,A,G_{B}\right),$$

where $L_{register}(A,B,G_{A})$ = $L_{similarity}\left(B,A◦\phi_{AB}\right)$ + $\lambda L_{smooth}(\phi_{AB})$; $L_{register}(B,A,G_{B})$ = $L_{similarity}\left(A,B◦\phi_{BA}\right)$ + $\lambda L_{smooth}(\phi_{BA})$, λ is the hyperparameter.

The functional MRI registration loss includes two parts: the functional MRI similarity loss and the local functional connectivity loss. In this study, the deformation field of the functional MRI is obtained by downsampling the corresponding structural MRI deformation field by 1/3($\phi_{\frac{1}{3}}$). The functional MRI similarity loss uses the MSE function.

(13) $$L_{similarity}(I_{fixed},I_{moving}◦\phi_{\frac{1}{3}})=MSE(I_{fixed},I_{moving}◦\phi_{\frac{1}{3}})\;=\frac{1}{\left|\Omega \right|}\sum_{p\in \Omega }[I_{fixed}\left(p\right)-[I_{moving}◦\phi_{\frac{1}{3}}](p){]}^{2},$$

Local functional connectivity loss of the functional MRI calculates the Euclidean distance of the local functional connectivity pattern between the registered and the fixed image. According to the local functional connectivity patterns in the method, the feature vectors $f({(I}_{moving}◦\phi_{\frac{1}{3}})(p))$, $f\left(I_{fixed}\left(p\right)\right)$, (p∈Ω) of the local functional connectivity patterns are obtained. We then compute the Euclidean distance between the two feature vectors as the functional similarity loss.

(14) $$L_{local_{\_func}}\left({(I}_{moving}◦\phi_{\frac{1}{3}},I_{fixed}\right)=\frac{1}{\left|\Omega \right|}\sum_{p\in \Omega }\left|\right|f\left({(I}_{moving}◦\phi_{\frac{1}{3}})\left(p\right)\right)-\;f\left(I_{fixed}\left(p\right)\right){\left|\right|}_{2},$$

where *p* is the voxel in the fMRI, and Ω denotes the fMRI spatial domain.

The total loss of functional MRI registration can be expressed as

(15) $$L_{register\_fmri}\left(X,Y,G_{A,},G_{B}\right)=L_{register}\left(X,Y,G_{A}\right)+L_{register}\left(Y,X,G_{B}\right),$$

where

(16) $$L_{register}\left(X,Y,G_{A}\right)=L_{similarity}\left(Y,X◦{\phi_{AB}}_{\frac{1}{3}}\right)+L_{{local}_{\_func}}\left(X◦{\phi_{AB}}_{\frac{1}{3}},Y\right)$$

(17) $$L_{register}\left(Y,X,G_{B}\right)=L_{similarity}\left(X,Y◦{\phi_{BA}}_{\frac{1}{3}}\right)+L_{{local}_{\_func}}\left(Y◦{\phi_{BA}}_{\frac{1}{3}},X\right)$$

**Figure 3:**
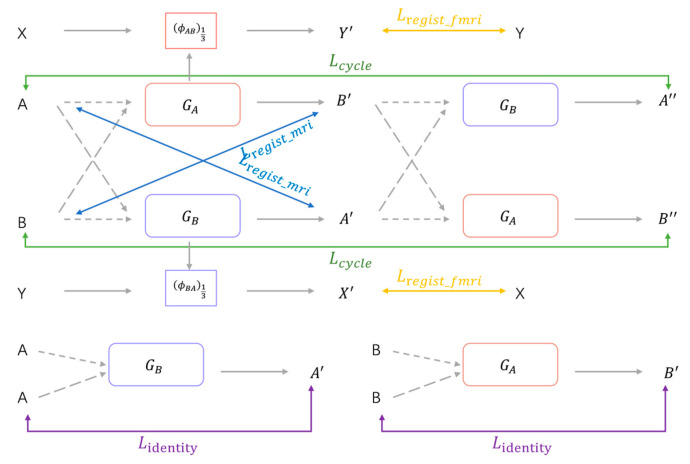
The diagram of the loss function structure in the proposed registration model.

In the registration, there is a cycle loss in this cycle consistent bidirectional registration structure. As shown in Figure 3, theoretically, after two successive deformations, the registered image should be able to return to the original image, that is, A ≅ $A^{\prime \prime }$; B ≅ $B^{\prime \prime }$. where $A^{\prime \prime }$ = T($B^{\prime },\phi_{BA}^{\prime })$; $B^{\prime \prime }$ = T($A^{\prime },\phi_{AB}^{\prime })$; T denotes the spatial transformation function. The cycle loss is derived from the difference between the moving image and the image after two successive deformations and is used to preserve the topology between them. Therefore, this study imposes cycle-consistent constraints between image A and $A^{\prime \prime }$, B and $B^{\prime \prime }$. The cyclic loss is implemented as follows:

(18) $$L_{cycle}\left(A,B,G_{A},G_{B}\right)=||T(B^{\prime },\phi_{BA}^{\prime })-A|{|}_{1}+||T(A^{\prime },\phi_{AB}^{\prime })-B|{|}_{1},$$

where ($B^{\prime },A^{\prime }$) = (T($A,\phi_{AB}),T(B,\phi_{BA}))$.

Considering that when the input moving image and the fixed image are the same, the registration network should not implement deformation of the moving image, background regions in the image should not be transformed when deforming the moving image during registration. If the input moving image is the same as the fixed image or for the background region in the moving image, the registration network should not generate deformation fields that are unreasonable in theory, and the same region of the registered image and the moving image should remain the same. Therefore, to constrain the registration network, in this case, to prevent unnecessary deformation, the identity loss is achieved in this study as follows:

(19) $$\begin{array}{c} L_{identity}\left(A,B,G_{A},G_{B}\right)\\ \;\;\;\;\;\;\;\;\;\;\;\;\;\;\;\;\;\;\;\;=L_{similarity}\left(B,{T(B,G}_{A}(B,B))\right)\\ \;\;\;\;\;\;\;\;\;\;\;\;\;\;\;\;\;\;\;\;+L_{similarity}\left(A,{T(A,G}_{B}(A,A))\right) \end{array}$$

Minimizing the identity loss maximizes the similarity between the registered image and the moving image, which encourages the registration network to perform no deformation for the same input image pairs. The identity loss improves the processing ability of the registration network for the same input image pair and improves the stability and accuracy of the registration network.

Combined with the above losses, the total loss of the unsupervised deep learning fMRI image registration model in this study can be defined as

(20) $$L\left(A_{mri},B_{mri},A_{fmri},B_{fmri},G_{A},G_{B}\right)\;=L_{register\_mri}\left(A_{mri},B_{mri},G_{A},G_{B}\right)+\alpha L_{register\_fmri}\left(A_{fmri},B_{fmri},G_{A,},G_{B}\right)\;+\beta L_{cycle}\left(A_{mri},B_{mri},,G_{A},G_{B}\right)+\gamma L_{identity}\left(A_{mri},B_{mri},,G_{A},G_{B}\right),$$

where $L_{register\_mri}$ denotes structural MRI registration loss, $L_{register\_fmri}$ denotes functional MRI registration loss, $L_{cycle}$ denotes cycle consistent loss, and $L_{identity}$ denotes identity loss. α, β, and γ are hyperparameters.

## 3. Experiments

### 3.1. Dataset

The dataset used in this study was obtained from the OpenfMRI database with the registration number ds000030 [40]. The dataset has a sample size of 273, with each subject varying in age, gender, and health status. T1-weighted anatomical imaging and bold contrast fMRI were selected for each subject in this study. We applied a series of standard preprocessing steps of motion correction, skull stripping, affine spatial normalization, and intensity normalization to each subject’s T1 anatomical imaging using FreeSurfer(v6.0.0), and cropped the resulting images. The resulting T1 anatomical image is of size 192 × 192 × 192 with 1 mm isotropic voxels. Moreover, we also performed anatomical segmentation on MRI to obtain label images containing 42 anatomical structures, each anatomical structure containing no less than 100 voxels. The dimensional size and isotropic voxel spacing of the label images were the same as the T1 anatomical images.

For each subject’s fMRI, preprocessing was performed using the DPARSF, including standard preprocessing steps such as removal of the first 10 time points, time-slice correction, head motion correction, and band-pass filtering with a filtering range of 0.01–0.1 Hz. All fMRI images were cropped to a size of 64 × 64 × 64 × 142, which is 1/3 of the size of a T1 MRI image, and resampled voxels with 3 mm isotropic. The method studied in this paper focuses on deformable registration between images. Therefore, the T1 MRI images and fMRI of the same subjects need to be affinely aligned before training. Specifically, the T1 MRI and fMRI of each subject were affine aligned to standard spatial templates with voxel dimensions of 1 mm and 3 mm, respectively, using SPM. By using visual inspection of the images for quality control, 244 valid subjects were finally obtained. In this study, the dataset was divided into training and testing sets with 220 subjects and 24 subjects, respectively, with each subject containing T1 MRI, resting-state fMRI, and anatomically segmented label.

### 3.2. Implementation Details

In the experiments, Pytorch was used as the backend to train the registration network on the NVIDIA RTX 3090 GPU(NVIDIA Corporation, Santa Clara, CA, USA.) The maximum number of iterations is set to 500 by default, using the ADAM optimizer with a learning rate of $10^{-4}$, the batch size set as 1, and the stochastic gradient descent method used to optimize the parameters. For the hyperparameter setting, the hyperparameters were set as α = 0.2, β = 0.1, γ = 0.5, and λ = 0.02 according to experience.

During the training process, the input T1-weighted image shape size in this study is (192, 192, 192), and the window size used by Swin Transformer is {6, 6, 6}. In the coding stage of the registration network, the number of Swin Transformer blocks is set to {2, 2, 4, 2} in order. The fMRI size is (64, 64, 64, 142), and the deformation field obtained from the registration of the T1-weighted image can be downsampled by 1/3 to obtain the deformation field required for the fMRI registration.

We conduct inter-subject image registration experiments to evaluate the proposed model. The specific implementation is that each subject is taken as a moving image, and one subject is randomly matched as a fixed image from the remaining dataset to form an image pair. We also made data enhancement on the dataset by flipping the image pairs to be registered, forming another image pair by switching the order of the moving and the fixation image. The final dataset consists of 440 and 48 image pairs for training and testing, respectively. As an unsupervised learning method, the registration model in this study does not use supervised information in both the training and testing stages.

### 3.3. Baseline Methods

This study compares the proposed fMRI registration methods with some baseline methods. Specifically, we select two traditional image registration methods (non-learning-based), Affine and Syn, and three learning-based image registration methods. Transmorph-tiny, VoxelMorph x2, and Cyclemorph, as the baseline methods.

Affine: The fMRI preprocessed in this study are all linearly aligned to MNI space through affine transformation, the fMRI are affinely aligned with each other. Therefore, the image pairs in the dataset have been initially affinely aligned to achieve linear registration.

SyN: ANTs(antspyx, 0.5.3) is the software with better quality medical image registration. SyN is an image registration algorithm in ANTs [41]. It is a non-learning-based method that does not require training. We utilize the python library ants; the version is 0.0.7. Specific parameter settings: SyNOnly registration type, MeanSquares is used as the similarity optimization metric in the symmetric normalization process, the iteration vector is set to (160, 80, 40), and Gaussian smoothing is used by default.

VoxelMorph x2: VoxelMorph is a classical and efficient deformable image registration method based on deep learning proposed in recent years that achieves the best registration performance [42]. In this study, VoxelMorph x2 (a variant of VoxelMorph, where “x2” refers to a model where the number of features is double) is used. The deformation field regularization parameter λ = 0.02 and the settings of the parameters are derived from the optimal performance of the baseline method.

Transmorph-tiny: Transmorph uses Transformer to establish remote spatial correspondences between image pairs in medical image registration firstly, expanding the limited receptive field of convolutional networks and providing multiple variants [43]. Due to the limitations of image volume and GPU memory, we use Transmorph-tiny as the registration model in the comparison experiments. The number of embedding dimensions is 6, and the number of Swin Transformer blocks is {2, 2, 4, 2}. The hyperparameters and loss function were set the same as the comparison experiment itself.

Cyclemorph: Cyclemorph is an improved image registration method based on VoxelMorph and a generative adversarial network. Their registration network uses the typical architecture of U-Net and has a better registration effect [37]. In the comparison experiment, the hyperparameters are set according to the parameters given by the experiment. Specifically, the cycle loss hyperparameter α = 0.1, the identity loss hyperparameter β = 0.5, and the deformation field regularization loss λ = 0.02.

### 3.4. Evaluation Metrics

The Dice score measures the degree of overlap between the anatomical structures of the images, and its value is in the range [0, 1]. The Dice score is 1 between the images that have perfectly matched anatomical structures. The more similar the corresponding structure between image pairs, the higher the Dice score is. For certain structures, the volume overlap between image B, warped by the deformation field, and the fixed image A can be calculated as follows.

(21) $$Dice\left(A^{s},B^{s}\right)=2\frac{\left|A^{s}\cap B^{s}\right|}{\left|A^{s}\right|+\left|B^{s}\right|},$$

The structural consistency between fixed and registered images was evaluated by calculating Dice coefficients for the corresponding anatomical segmentation maps to evaluate the registration performance of different registration methods.

The registration process drives the registered image to be more and more similar to the fixed image, which may lead to the deformation field producing spatial folding and loss of topology. Diffeomorphic registration has a smooth deformation field with a non-zero Jacobian matrix. In this study, the Jacobian matrix is used to quantitatively evaluate the folding degree of the generated deformation field during registration by calculating the percentage of negative values in the Jacobian matrix of the deformation field:

(22) $$\left|J_{\emptyset }\left(p\right)\right|=\left|\nabla \phi \left(p\right)\right|\leq 0,$$

where *p* denotes the non-background voxel position in the deformation field. A lower negative percentage of the Jacobian determinant of the deformation field indicates a smoother deformation field, resulting in fewer spatial folds and a better quality of the deformation field generated during registration.

There is no gold standard to evaluate the performance of fMRI registration in the current fMRI registration research, and most scholars have used assessment methods based on group-level statistical maps of brain functional networks [21,29,34,35]. Independent component analysis (ICA) can be used to extract brain functional networks by performing group ICA operation using GIFT software(v3.0b) to extract four common brain functional networks, which are default mode network (DMN), visual network (VN), central executive network (CEN), and sensorimotor network (SMN). The ICASSO method is selected to perform 100 ICA analyses, 20 independent components are obtained through reverse reconstruction, and then the above 4 brain functional networks are determined according to the relevant network templates. Based on the extracted four brain functional networks, the functional consistency between subjects was further evaluated.

Then, this study used three analysis criteria to evaluate the functional consistency of different registration methods: the co-activation of four brain functional networks in the group-level T-map, the peak-t value of the t-map, and the number of suprathreshold significant voxels. For each subject in the dataset to be evaluated, four functional brain networks were extracted, and one-sample t-test was performed on the four functional brain networks of a group of subjects to obtain the group-level t-map and the peak t values. The group of subjects with a greater degree of co-activation of the functional brain networks had a higher functional consistency and higher peak t values of the corresponding t-map. For the comparison of the number of suprathreshold significant voxels, the number of suprathreshold voxels for each network was calculated given a specific threshold value of t = 5.01 (*p* < 0.05). The better the registration, the higher the number of suprathreshold voxels in the corresponding brain functional network.

## 4. Results

In most studies, there are usually two main approaches to image registration: atlas-based registration and inter-subject registration. Atlas-based registration is a common approach in group analysis, and inter-subject registration is the central issue of group analysis [42]. Compared with the atlas-based registration, inter-subject registration has better generality. To evaluate the method proposed in this study, several comparative methods, Affine, SyN, Transmorph-tiny, VoxelMorph x2, and Cyclemorph, were selected to perform inter-subject registration. In comparing these methods, the loss function uses the same mean square error (MSE) calculation method except for the different hyperparameters.

Table 1 shows the quantitative evaluation results of inter-subject registration on the test dataset for all registration methods. The metrics include the Dice scores, the percentage of negative Jacobi matrix values for the deformation field, and the test running time. In terms of Dice performance, the method we proposed in this study is comparable to ANTs; outperforms Transmorph-tiny, VoxelMorph x2, and Cyclemorph; and significantly outperforms affine registration. In terms of the smoothness of deformation fields, the deformation fields generated by the registration model in this study are smoother than those generated by other learning-based methods with a lower degree of folding. This shows that our registration model easily preserves the topology of the deformation field and ensures better smoothness of the deformation field while improving the registration effect. In terms of runtime, since it is not necessary to optimize each test image pair, the proposed method and other learning-based registration methods require less inference running time, and the testing process can be completed within 1 s using the general registration model obtained in the training stage. However, the traditional registration method ANT needs to optimize each test image pair, and the inference running time is longer. In general, among the compared methods, the method we proposed in this study not only improves the Dice score and improves the image registration accuracy but also reduces the folded voxels of the Jacobian matrix in the deformation field, which is the outstanding-performing method in the structural consistency of fMRI registration.

**Figure 4:**
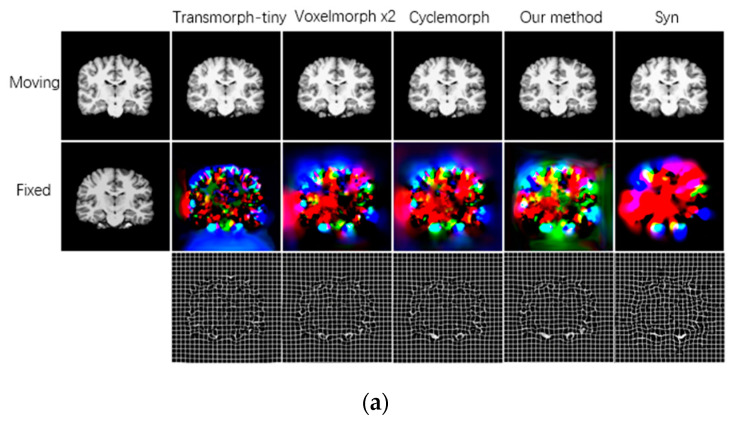

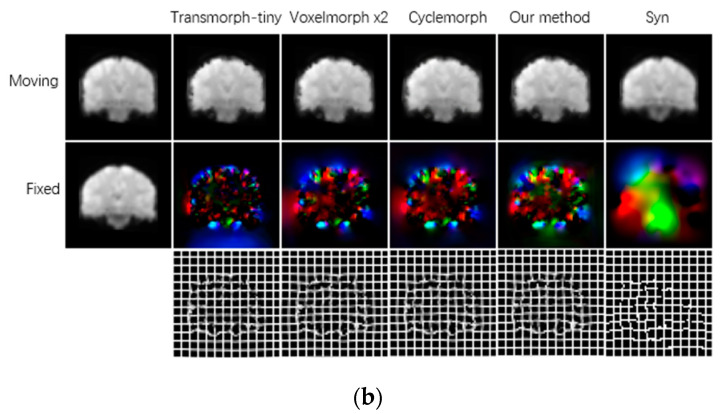
Visualization of inter-subject image registration results for different registration methods on the test dataset. (**a**) visualization on the MRI registration; (**b**) visualization on the fMRI registration. For a pair of moving and fixed images, the first row on the right shows the registered image obtained by warping the moving image with different registration methods, the second and third rows show the RGB and grid visualization of the deformation field.

The qualitative evaluation results of structural consistency with all registration methods are shown in Figure 4. The learning-based approach produces a more accurate deformation field compared to the traditional image pair optimization method and performs an exact deformation of the moving image to register it to the fixed image. Due to the bidirectional registration and cycle consistency constraints, the method in this study produces smoother deformation fields relative to other comparative methods, which also indicates that the proposed method in this study better preserves the topology of the deformation field and has a higher quality of image deformation.

For the evaluation of functional consistency between the registered fMRI images, this study conducted an atlas-based registration experiment, where one subject was selected from the test dataset as the atlas, and the other subjects in the test dataset were used as moving images to form an image pair with the selected atlas. All moving images were uniformly registered to the atlas, resulting in a set of fMRI data registered to the atlas.

**Figure 5:**
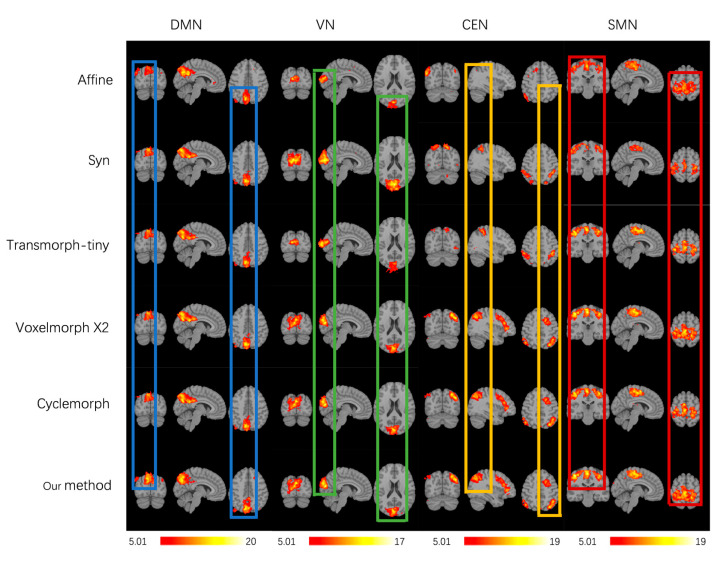
Group-level t-maps of four functional brain networks (DMN, VN, CEN, SMN) with different registration methods, t > 5.01 (*p* < 0.05). Different colored vertical rectangular boxes represents the cropped regions with significant differences in the degree of co-activation on functional networks, e.g. blue region is cropped from DMN.

**Figure 6:**
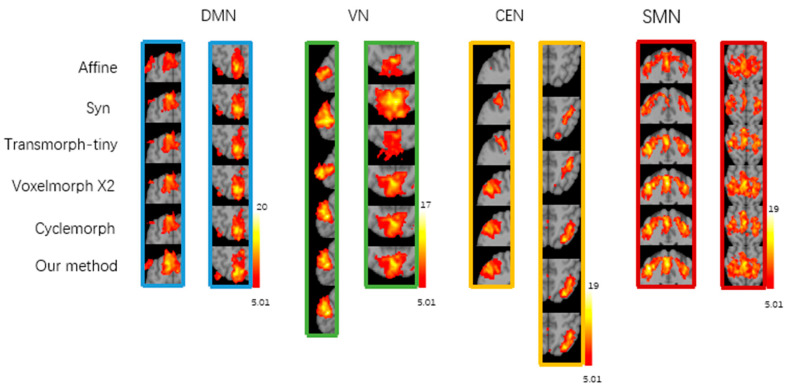
Local magnification of co-activated regions in group-level t-maps of four functional brain networks (DMN, VN, CEN, SMN) with different registration methods, t > 5.01 (*p* < 0.05). Different colored vertical rectangular boxes represents the local magnification of the cropped regions with significant differences in the degree of co-activation on functional networks, e.g. blue region is cropped from DMN.

Figure 5 shows the group-level t-maps obtained by one-sample t-test from the four brain functional networks of registered fMRI with different registration methods, where t > 5.01 (*p* < 0.05). After correlation regression and visual inspection, the average independent components extracted from a set of fMRI images were reasonably divided into four brain functional networks, namely, the default mode network (DMN), visual network (VN), central executive network (CEN), and sensorimotor network (SMN). The group-level t-maps show the degree of voxel co-activation on four brain networks obtained from a group of subjects with different registration methods. The more voxels co-activated on the functional brain networks, the larger the co-activated regions, the larger the co-activation value, and the brighter the co-activated regions, indicating better functional consistency of this group of functional images. As shown in Figure 6, from the local magnification of the co-activation region in the group level t-map, it can be seen that the registration method in this study shows a larger voxel co-activation region and a brighter co-activation region in the DMN, CEN, and SMN brain networks. The Syn method has a larger voxel co-activation area in the VN brain network. Therefore, compared with the baseline registration method, the functional consistency between a group of functional images registered by our proposed registration method is higher.

Table 2 shows the peak t-value in the t-maps of DMN, VN, CEN, and SMN brain functional networks obtained by different registration methods. Our registration method significantly increased the peak t-value of DMN, CEN, and SMN brain functional networks, where the peak t-value increased to 18.7 in the DMN network, 16.6 in the CEN network, and 17.3 in the SMN network; the Syn method significantly increased the peak t-value in the VN brain functional network, where the t-peak increased to 22.9. Compared with Affine, Syn, and learning-based registration methods Transmorph-tiny, Cyclemorph, and VoxelMorph x2, our method has improvements of 23.79%, 12.74%, 12.27%, 7.32%, and 5.43%in the peak t value of t-maps.

We also evaluated the number of suprathreshold voxels for different registration methods with a given threshold t > 5.01 (*p* < 0.05, FDR corrected), as shown in Table 3. Given the threshold, the registration method proposed in this study has the largest number of super-threshold voxels in DMN, CEN, and SMN brain functional networks, which are 2085, 2978, and 2986, respectively. Syn had the largest number of super-threshold voxels in the VN brain network, consistent with the group-level t-map results. In the learning-based baseline methods, the number of suprathreshold voxels of DMN, VN, CEN, and SMN is consistent with the results of structural consistency. The improvement in the functional consistency of fMRI registration based on structural images compared to affine depends on the performance of structural consistency. The higher the Dice for structural consistency, the better the structural registration, and the corresponding functional consistency registration improves. According to the number of suprathreshold voxels on different functional networks, for methods with a general structural consistency registration, for instance, Transmorph-tiny has a lower number of suprathreshold voxels. The difference in the methods that are more effective in structural consistency registration is obvious, and sometimes doubled difference, such as in VN and CEN networks. The statistical number in Table 3 indicates that the registration method proposed in this study can maximize the number of super-threshold voxels in the group level t-map of the group of functional image groups after registration, effectively improving the functional consistency between the registered fMRI images, and the registration effect is better.

## 5. Discussion

Registration between individuals is an important step in fMRI preprocessing and the basis for subsequent fMRI studies. In previous fMRI image registration studies, the correspondence between fMRI images can refer to structural correspondence or functional correspondence, and the definition criteria are not clear. Traditional fMRI registration assumes that fMRI registration with the help of MRI registration achieves functional correspondence along with structural correspondence. However, it has been shown that anatomical structures do not correspond strictly and consistently with functional regions [8].

Currently, deep-learning-based methods are gradually being used for registration tasks with better registration performance and much faster computation than the traditional registration methods based on iterative optimization between image pairs. Compared with the unidirectional registration approach, the deformation field generated by the cycle-consistent bidirectional network we proposed has better reversibility and smoothness. While improving the registration performance, the registration model of the proposed method preserves the topology of the deformation field well. In this study, Transformer is used in the registration network to establish the remote spatial correspondence between images, effectively improve the limitation of the limited receptive field of the convolutional neural network, and enhance the feature extraction ability of the registration network.

This study conducted inter-subject registration during training and evaluated atlas-based registration during testing. In terms of the speed of registration, the registration method proposed in this study, which trains the optimal global parameters on the training set, can provide fast and accurate image registration for unseen image pairs. It takes less than 1 s to register a pair of images on GPU, which greatly improves the registration speed compared with traditional non-learning-based registration methods such as ANT and significantly accelerates the speed of medical image registration. In terms of registration performance, the experimental results show that compared with the traditional registration method ANT and the currently effective learning-based methods Transmorph-tiny, VoxelMorph x2, and Cyclemorph, the proposed method in this study achieved a higher Dice score between the registered image and the fixed image compared to the learning-based comparison method and a comparable Dice score compared to the ANT registration method. Meanwhile, the registration method in this study generated a lower percentage of non-positive Jacobian matrix in the deformation field, indicating that the registration method in this study can provide topology-preserving image deformation between the moving and the registered image and generates a smaller number of deformation field voxels folded, which preserves a good topology. For the functional consistency assessment, the different methods were evaluated by generating group-level t-maps using a one-sample t-test on the four brain functional networks. Our registration method showed larger co-activation regions, higher peak t-value, and a larger number of suprathreshold voxels for a given threshold case in the DMN, CEN, and SMN brain networks compared to the comparison methods. These indicate that our registration method achieves better inter-subject functional consistency between the registered images.

Due to the model architecture design, for instance, our method employs bidirectional registration, trains four registration networks simultaneously with Transformer, and incorporates functional loss functions, making the model more structurally complex and thus affecting the computational complexity. We will be devoted to further improving our model to reduce computational resource consumption and increase efficiency in the future.

## 6. Conclusions

Traditional fMRI registration based on structural images fails to achieve good functional consistency between subjects. This paper proposes an unsupervised deep learning model for a non-rigid cycle consistent registration bidirectional registration network with Transformer for deformable fMRI registration. The performance improvement was achieved mainly from three aspects: improving the registration accuracy, preserving the topology of the deformation field, and improving the functional correspondence of fMRI. On the basis of fMRI registration based on structural information, this study added functional information about fMRI during the training of the registration model and designed the local functional connectivity loss function to compute the functional similarity loss between the registered and fixed images to improve the functional consistency between the registered images. On the whole, our proposed registration model has a cycle-consistent bidirectional registration structure using two registration networks, forward and reverse, to realize bidirectional registration between image pairs to train the registration network. While improving the registration performance, the registration model of the proposed method preserves the topology of the deformation field well. Experimental results demonstrate the effectiveness of the proposed fMRI registration method, which outperforms various traditional methods and learning-based registration methods in terms of structural consistency and functional consistency and achieves superior registration performance.

## Figures and Tables

**Table 1:** Quantitative evaluation results of inter-subject registration on different registration methods, showing Dice score, percentage of negative values of folded voxels of the Jacobian matrix, and running time. The runtime represents the average inference time for a pair of image pairs, where SyN runs on the CPU and all other learning-based methods are implemented on the GPU.

Method	DSC	$\%\;\mathrm{of}\;|J_{\emptyset }|\leq 0$	Inference (s/Pair)
Affine	0.487 ± 0.069	-	-
Syn	0.716 ± 0.050	<0.0001	223.709
Transmorph-tiny	0.620 ± 0.059	0.00519	0.196
VoxelMorph x2	0.692 ± 0.045	0.00163	0.206
Cyclemorph	0.683 ± 0.046	0.00128	0.198
Our method	0.722 ± 0.044	0.00127	0.223

**Table 2:** Peak t values of group-level t-maps on DMN, VN, CEN, and SMN brain functional networks with different registration methods.

Method	DMN	VN	CEN	SMN
Affine	16.9	13.8	12.4	13.2
Syn	17.3	22.9	13.5	11.7
Transmorph-tiny	17.7	15.2	13.8	15.1
VoxelMorph x2	18.5	15.9	14.5	16.9
Cyclemorph	18.2	15.6	14.4	16.4
Our method	18.7	16.5	16.6	17.3

**Table 3:** Number of suprathreshold voxels of DMN, VN, CEN, SMN with different registration methods, t > 5.01 (*p* < 0.05).

Method	DMN	VN	CEN	SMN	Average
Affine	1834	841	1292	2505	1618
Syn	1868	2799	1410	1836	1978.25
Transmorph-tiny	1891	1218	1333	2637	1769.75
VoxelMorph x2	1984	2295	2790	2910	2494.75
Cyclemorph	1925	2197	2481	2803	2351.5
Our method	2085	2336	2978	2986	2596.25

## Data Availability

The dataset was obtained from a large study initiated and implemented by the UCLA Consortium for Neuropsychiatric Phenomics LA5c Study (Bilder R. 2016. OpenfMRI. Ds000030): https://openfmri.org/dataset/ds000030/ (16 December 2024).
